# A simplified study of trans-mitral Doppler patterns

**DOI:** 10.1186/1476-7120-6-59

**Published:** 2008-11-28

**Authors:** George Thomas

**Affiliations:** 1 Department of Cardiology, Saraf Hospital, Sreekandath Road, Kochi 682016, India

## Abstract

**Background:**

Trans-mitral Doppler produces complex patterns with a great deal of variability. There are several confusing numerical measures and indices to study these patterns. However trans-mitral Doppler produces readymade data visualization by pattern generation which could be interpreted by pattern analysis. By following a systematic approach we could create an order and use this tool to study cardiac function.

**Presentation of the hypothesis:**

In this new approach we eliminate the variables and apply pattern recognition as the main criterion of study. Proper terminologies are also devised to avoid confusion. In this way we can get some meaningful information.

**Testing the hypothesis:**

Trans-mitral Doppler should be seen as patterns rather than the amplitude. The hypothesis can be proven by logical deduction, extrapolation and elimination of variables. Trans-mitral flow is also analyzed *vis-à-vis *the Starling's Law applied to the left atrium.

**Implications of the hypothesis:**

Trans-mitral Doppler patterns are not just useful for evaluating diastolic function. They are also useful to evaluate systolic function. By following this schema we could get useful diagnostic information and therapeutic options using simple pattern recognition with minimal measurements. This simplified but practical approach will be useful in day to day clinical practice and help in understanding cardiac function better. This will also standardize research and improve communication.

## Background

Echocardiography has become an important diagnostic tool in clinical cardiology. With the advent of Doppler studies, analysis of trans-mitral left ventricular Doppler filling patterns (LV DFP) has assumed importance.[1] LV DFPs are commonly used to study diastolic function.[2] Details of assessment of diastolic function are given elsewhere.[3] These are complex patterns with a great deal of variability. There are several confusing measures and indices to study these patterns. However, by following a systematic approach and using proper terminology we could create an order and use this tool to study systolic function also. This new approach to trans-mitral Doppler patterns could be useful in practice for the study of cardiac function by pattern recognition and help in planning optimum therapy.

When the scientific world is betting on the power of human visual intelligence to find patterns in data, it is unfortunate that these readymade transmitral Doppler patterns are not used as such. Instead the present methodologies reconvert these useful patterns to numerical data. The value and extraordinary power of data visualization for analysis is quickly gaining recognition. This should also be used in the analysis of Doppler patterns.

## Presentation of the hypothesis

Trans-mitral Doppler interrogation produces a wide range of patterns. But the predominant feature is a biphasic flow. The early flow is the E wave and the late flow is the A wave. Occasionally a third wave may be noted between the two. This coincides with the partial opening of the mitral valve following the M-mode F point. So this wave may be termed G wave. In this discussion we are only considering flow Doppler patterns. The author is of the view that there are methodological flaws in tissue Doppler.[4] Hence these are not included in the analysis. Besides, incorporating this modality would complicate matters going against our aim of simplifying the subject.

The highly variable nature of these patterns makes them difficult for linear analysis. In this new approach we eliminate the variables and apply pattern recognition as the main criterion of study. Proper terminologies are also devised to avoid confusion. As a first step in this elimination process, this method is used in normal valves only. By normal valves we also exclude valve stiffness, which could affect Doppler patterns. An example is mitral valve stiffness not amounting to stenosis.

There is a common understanding that trans-mitral Doppler patterns are used to study diastolic function. However, these patterns should be considered as a 'tool' which just happens to occur in diastole. Then we can have a wider perspective and use the 'tool' to study systolic function too. Hence these patterns are called 'filling patterns' without reference to the cardiac cycle. The inferences derived from the Doppler pattern depend on the systolic function. It is like using the measuring tape to measure abdominal girth in a female. The inference from the measurement will depend on whether the patient is pregnant. It is a case of same tool, similar measurement but different inference depending on the clinical situation.

The various numerical indices used to study these patterns have been complex and confusing for routine clinical practice. All these indices are dependant on several variables like chamber properties, pressure changes, heart rate etc. However by pattern recognition these can be simplified so that useful information can be derived in day-to-day practice. Thus 2 types of information can be derived from LV DFP. 1. Chamber properties 2. Hemodynamic information.

### LV Chamber properties

**Figure 1:**
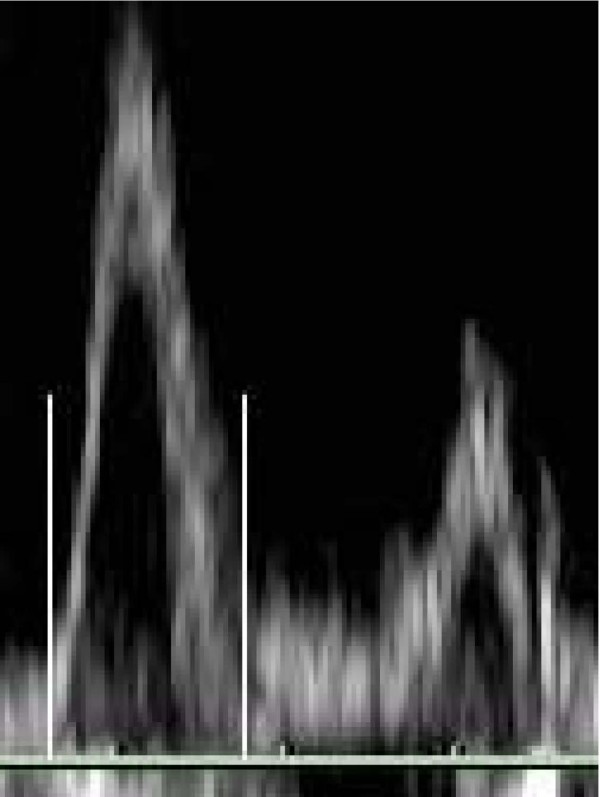
**Measurement of Early Filling Time (EFT)**. Figure showing the measurement of the Early Filling Time. This is measured from the beginning of the E wave to the end. This measurement will be rarely required once the operator gets used to the machine sweep setting and keeps it constant.

**Figure 2:**
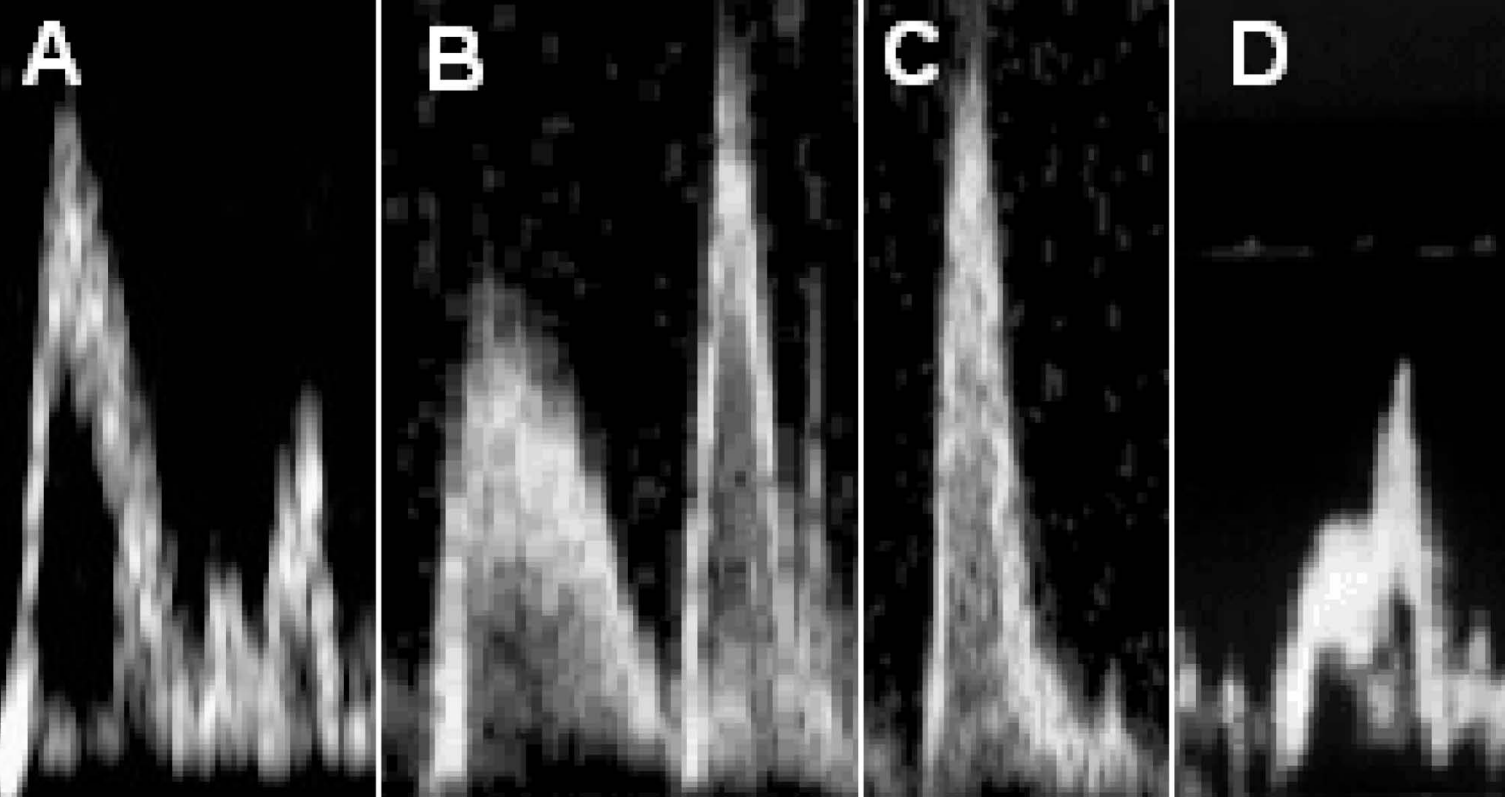
**Different recognizable patterns in a patient with normal systolic function**. Panel A shows the normal pattern. Panel B shows the 'R pattern'. This pattern has a prolonged EFT and usually shows E-A reversal. Panel C shows the 'C pattern'. This pattern has a shortened EFT. Panel D shows an indeterminate pattern with E and A merging. The normal pattern in panel A could also be an 'RC pattern' a combination of the R and C patterns. This can only be identified based on the clinical circumstance.

LV DFP can be used to study the chamber properties. This is more relevant when the systolic function is normal (see below). Here 2 recognizable patterns are discernable. 1. Relaxation abnormality pattern or 'R pattern' 2. Compliance abnormality pattern or 'C pattern'. Many indices are used for chamber properties. However the commonly used index is E wave deceleration time. There are practical problems in determining the deceleration time. For example with the available software it is difficult to accurately determine the deceleration time in a curved "ski-slope" Doppler tracing. A simpler and more accurate method would be determination of the early filling time (EFT). This is the duration of the E wave from the beginning of the E wave to the end (see figure 1). A prolonged EFT (>250 milliseconds) could point to relaxation abnormality (R pattern) and a shortened EFT (<150 milliseconds) could point to a compliance abnormality (C pattern). In some cases where the E and A waves merge, it would not be possible to measure the EFT. In such cases the pattern would be 'indeterminate' (see figure 2). A normal looking combination of the 2 patterns is to be called RC pattern. The R pattern, RC pattern and C pattern are to be distinguished from patterns 1, 2 and 3 (see below). To avoid confusion it is important to term this aspect of the study as 'chamber properties' rather than 'diastolic function'.

### Hemodynamic information

**Figure 3:**
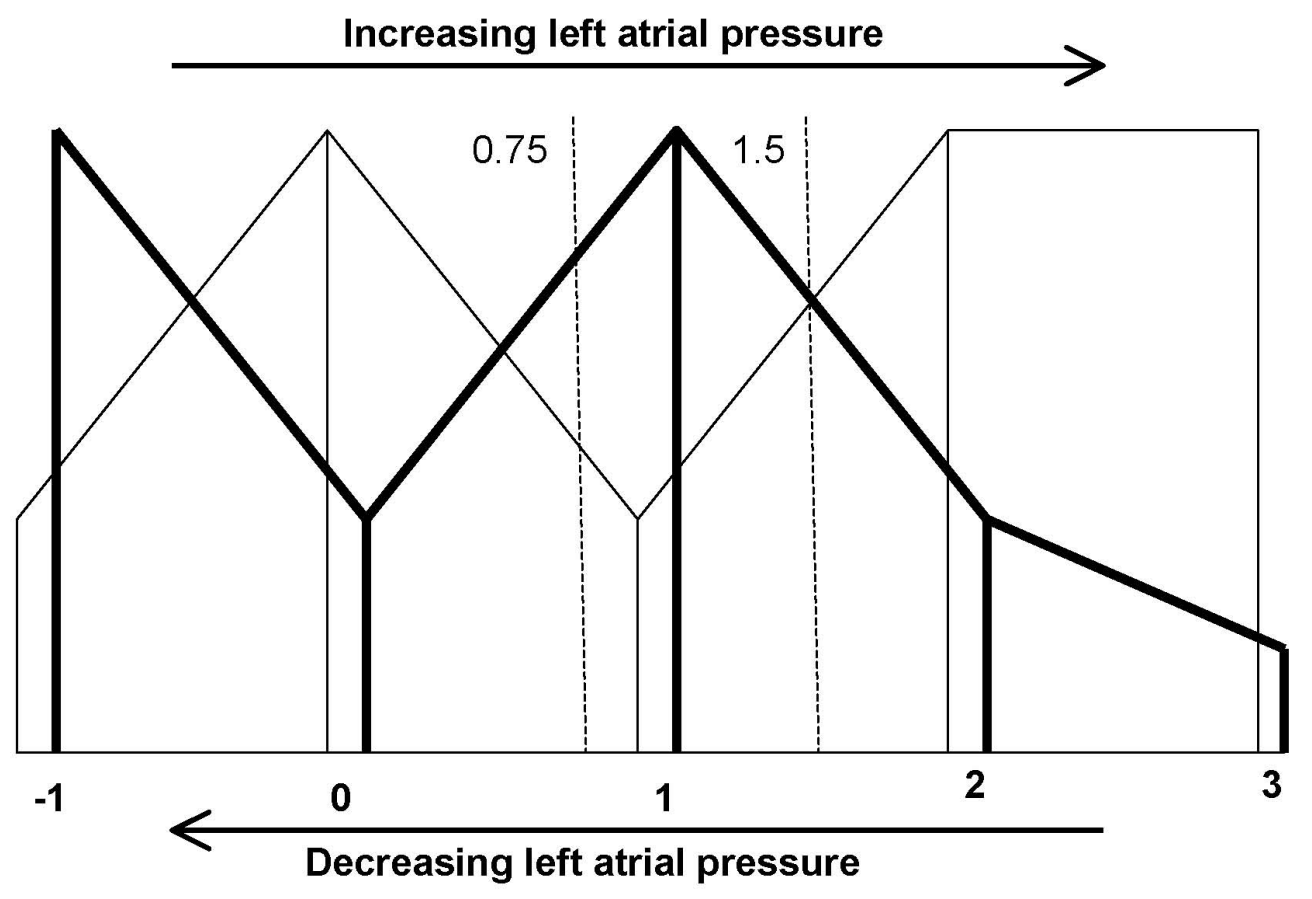
**Schematic representation of the variations in Doppler filling patterns in relation to the left atrial pressures**. The thick vertical lines represent the A waves and the thin lines represent the E waves. The main patterns from -1 to 3 are shown. Note that these are not absolute velocities but represent relative heights of the waves. The relative behavior of A and E waves with relation to left atrial pressures is extrapolated by the connecting thick and thin lines. This is a continuous variable and we can determine the pressure by determining the pattern at the appropriate point on the horizontal axis. A shift to the right indicates increasing pressure and a shift to the left indicates decreasing pressure. It is also possible to accurately label a particular pattern. For example E = A could be pattern -0.5, 0.5 or 1.5 and if E is lesser than A, the pattern could be -0.75, 0.75 or 1.25. The exact pattern could then be determined by the clinical circumstance and/or using a physiological maneuver and/or drug challenge. These hierarchal changes are best demonstrated in the young especially with systolic dysfunction.

LV DFP concurrently provides hemodynamic information (see figure 3). LV DFP is dependant on left atrial pressures (LAP). Variations in LAP can be studied by the behaviour of the E and A wave *relative *velocities. Based on this 5 main patterns can be recognized. LAP is a continuous variable so a number of intermediate patterns are possible. The main patterns are termed -1, 0, 1, 2, and 3. Using the current terminology, pattern 0 is 'normal' with E>A, pattern 1 is 'E/A reversal', pattern 2 is the 'pseudo-normal', and pattern 3 is the 'restrictive'. Pattern -1 is also E/A reversal but due to decreased pre-load as seen in dehydration.[5] The advantage is that we can describe any pattern with this numerical system. Thus pattern 1.5 will be E = A with high LAP. Increasing preload shifts a pattern to the right and conversely decreasing preload shifts a pattern to the left.[6,7] A shift to the right will indicate increasing LAP while a shift to the left will indicate decreasing LAP. Pattern shift to the left or right is possible with physiological maneuvers or pharmacological interventions that decrease or increase the LAP.[8] When the LAP is markedly elevated it may not be possible to shift pattern 3 to the left. This is the irreversible pattern and should be termed 3+. In the case of hemodynamic information it is better to use the precise numerical terms rather than terms like 'restrictive' or 'pseudo-normal'.

Any pattern can be accurately described with this classification and terminology. As a general rule any pattern can be described as hemodynamic pattern plus the chamber property. For example in a patient with a relaxation abnormality and normal systolic function the pattern could be reported as 1 R. In situations where there is variability, each pattern can be individually described or the modal pattern should be considered.

### Clinical significance of Doppler patterns

To understand the significance of LV DFP we need to consider 2 situations. 1. In patients with normal LV systolic function and 2. In patients with impaired systolic function.

### DFP in normal systolic function

In cases with normal systolic function, it is primarily a study of diastolic function ('diastology'). Here LV DFP could have diagnostic roles. In these cases of 'pure diastolic dysfunction' the 'impaired relaxation' or R pattern could point to hypertension, diabetes, coronary artery disease or hypertrophic cardiomyopathy. It could also signify age related changes.[9] The 'compliance abnormality' or C pattern could mean restrictive cardiomyopathy or pericardial disease. It is also seen in young adults. There could be a combination of R and C patterns resembling a normal pattern. This should be designated as RC pattern. In the case of normal systolic function the individual parameters may lose their validity.[10] So unlike systolic dysfunction these are distinctive patterns and there is no hierarchy of patterns. In terms of hemodynamics the patterns could be anywhere between -1 and 1 indicating an LAP of 9–15 mmHg.

### DFP in impaired systolic function

In cases with impaired systolic function, it is primarily study of systolic function ('systology'). Systolic dysfunction is usually associated with increased LV end diastolic pressure and left atrial pressure.[11] This can be studied by LV DFP.[12] Basic echocardiography tells us that a brisk and well opening mitral valve signifies good systolic function. Thus diastolic phenomena can be used to assess systolic function. Patients with progressively more abnormal Doppler patterns have greater structural abnormalities with larger left atrial and LV size and lower LV ejection fractions.[13] So, in cases with impaired systolic function, LV DFP can be used to assess the severity of hemodynamic impairment and prognosis. The information obtained is distinctive of systolic function and should not be confused with 'diastolic dysfunction'. In such cases trans-mitral Doppler shows a continuous pattern of changes (hierarchy of patterns) depending on the severity of hemodynamic impairment (see 'hemodynamic information', above). Here again it is better to consider these patterns as indicative of the severity of *systolic dysfunction *rather than severity of diastolic dysfunction (see below). By impaired systolic function we could also include cases with wall motion abnormalities with *apparently *normal ejection fraction and apparently normal overall LV contractility. Pattern 1 shows a small E wave and a large A wave could indicate mild hemodynamic impairment. Pattern 2, shows a 'pseudo-normalization' pattern denoted by an apparently normal E and A wave. Pattern 2 could indicate moderate impairment. Finally, pattern 3 is a very prominent E wave and a miniscule A wave. Pattern 3 could indicate severe hemodynamic impairment and is associated with the worst prognosis.[14] In cases of systolic dysfunction, the LV DFP is a continuous variable reflecting the increasing left atrial pressures which proceeds to atrial failure in pattern 3. As a rule of thumb patterns 0–1 signifies a pressure of about 12–15 mmHg, patterns 1–2 about 15–18 mmHg, patterns 2–3 about 18–20 mmHg and pattern 3+ greater than 20 mmHg. These pressure values are only indicative. The pattern recognition and its changes are best in the same patient with a reasonably maintained heart rate. With this approach the absolute velocity values become irrelevant. The relative E and A magnitudes will allow pattern recognition. The increasing left atrial pressure could be confirmed by the pulmonary venous flow abnormalities in ideal conditions. As a corollary, in cases with resting or stress induced wall motion abnormalities and apparently normal ejection fraction, a pattern to the right of 0 could indicate a systolic dysfunction.[15] In serial studies of chronic cases, a shift to the right could indicate worsening systolic function while a shift to the left could indicate improvement. In all these cases a reasonably preserved atrium is assumed.

### DFP in combined diseases

Diseases with combined systolic dysfunction and abnormal chamber properties will show a combination of the above features. For example, in a case of systolic dysfunction with a pattern 1.5 if there is prolonged EFT then the pattern could be described as 1.5 R.

**Figure 4:**
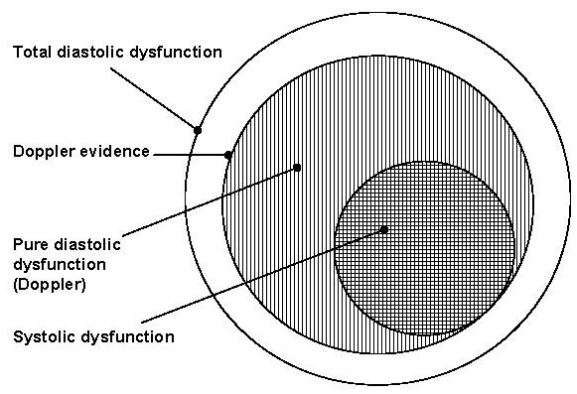
**Venn diagram showing components of cardiac dysfunction**. The largest circle shows the total diastolic dysfunction. The Doppler assessed diastolic dysfunction is shown by the vertically hatched middle circle and the pure diastolic dysfunction excludes the horizontally hatched inner circle. The Doppler patterns have different implications in pure diastolic dysfunction and systolic dysfunction. Note that the inner circle of systolic dysfunction has elements of diastolic dysfunction too. But in clinical situations it is better to view this part as 'systolic dysfunction' for treatment purposes. Any cardiac pathology has the potential to produce 'diastolic dysfunction' hence in clinical practice the use of the term is meaningless.

In this context it is appropriate to discuss the much abused term 'diastolic dysfunction'. Diastole is a sensitive part of the cardiac cycle. The earliest abnormality in any cardiac pathology is diastolic dysfunction. So it is but natural that *any *cardiac disease can cause LV diastolic dysfunction to *variable *extents (see figure 4). Doppler can only detect LV diastolic dysfunction at later stages. Abnormal DFP is only a part of the total diastolic dysfunction set. So being a ubiquitous entity, absence of abnormal DFP need not mean absence of diastolic dysfunction. LV diastolic dysfunction can occur in very mild forms and regional forms, which may not be evident on Doppler assessment. So instead of using the vague term 'diastolic dysfunction' it would be better to use specific terms as mentioned as described earlier. Heart being a pump, the systolic function is critical. The vague entity of diastolic dysfunction should not divert our attention from assessing the important systolic dysfunction as there are distinct treatment options for systolic dysfunction.

## Testing the hypothesis

Analysis of LV DFP is complicated. Technical factors play an important role in the acquisition and interpretation of the Doppler signals.[16,17] Several factors like load and heart rate affect Doppler parameters.[18] Even in load and heart rate independent methods, there is a broad overlap in Doppler filling parameters for normal and abnormal subjects. DFPs vary with respiration [19] and changes in position. There is also an inability to apply most methods in atrial fibrillation and other rhythm and conduction disturbances.[20] Coexisting cardiac conditions like valvular lesions and drug treatments complicate the issue even further. Doppler patterns reflect the cardiac physiology at the particular instant and display significant variability. There are some gray areas too. Considering these problems it would be better to look at the progression of Doppler patterns rather than the amplitude. The hypothesis can be proven by approaching the whole concept of transmitral flow *vis-à-vis *the Starlings Law.

**Figure 5:**
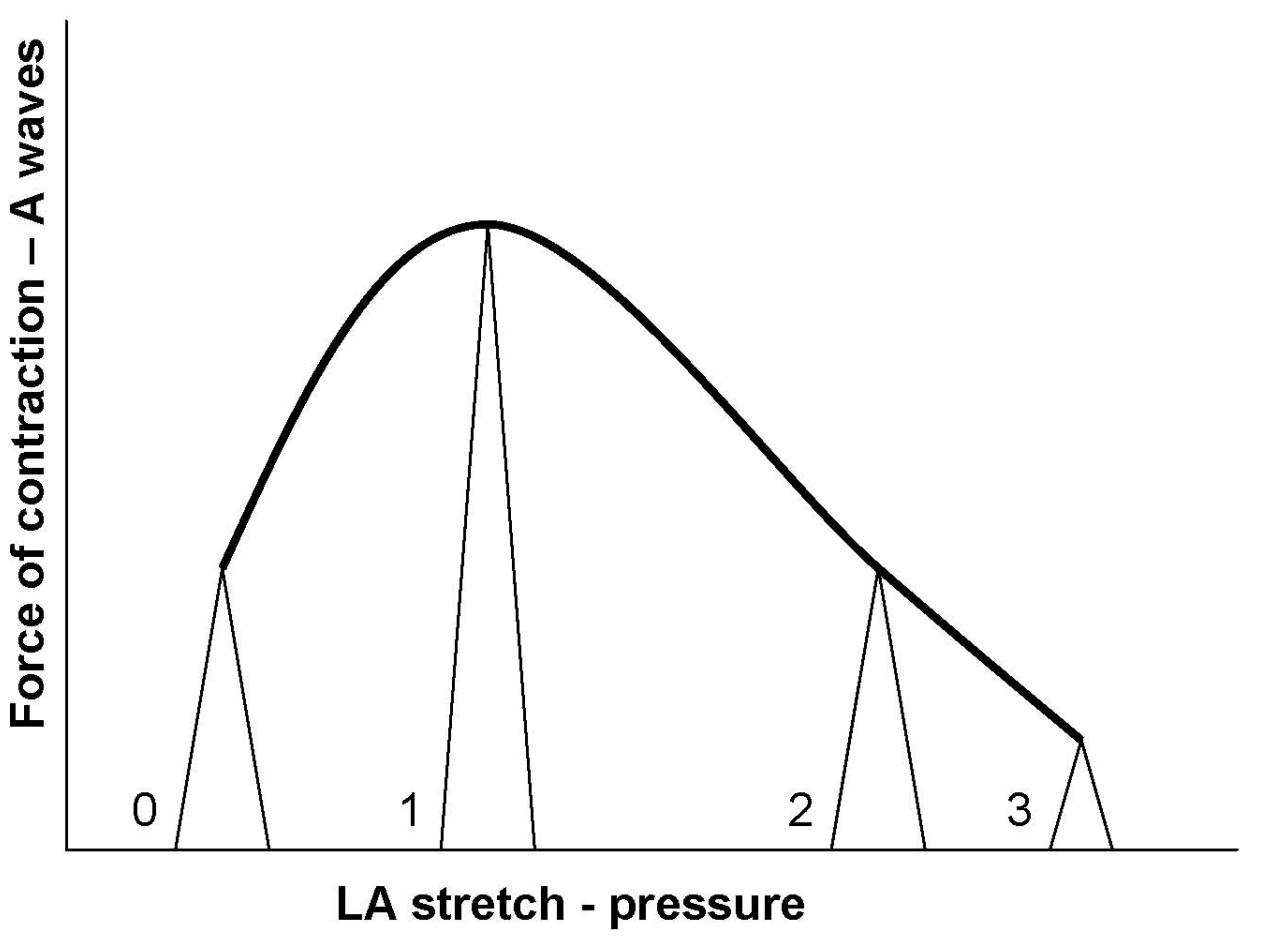
**Diagram showing the Starlings law applied to the left atrium**. The force of contraction and A wave magnitude is represented on the vertical axis and the left atrial stretch due to increasing left atrial pressure is represented on the horizontal axis. The different A wave patterns are superimposed on the Starlings curve. The force of contraction (A waves) increases with increasing stretch (left artial pressure) till physiological limit is reached at pattern 1. Beyond that there is progressive left atrial failure.

If we start with A wave, then in our hypothesis A is followed by E. So LV filling would start with atrial contraction and end with passive filling. The passive filling (E wave) will compensate for the atrial output. The progression of the A wave reflects the Starlings Law (see figure 5). The force of contraction increases with increasing left atrial pressure till the physiological limit is reached following which the force decreases. The E wave will follow the A wave in a reciprocal manner. Thus the behavior of A waves will reflect the left atrial pressure. The combined A and E appearances will be confirmatory.

### Diagnostic Methodology

**Figure 6:**
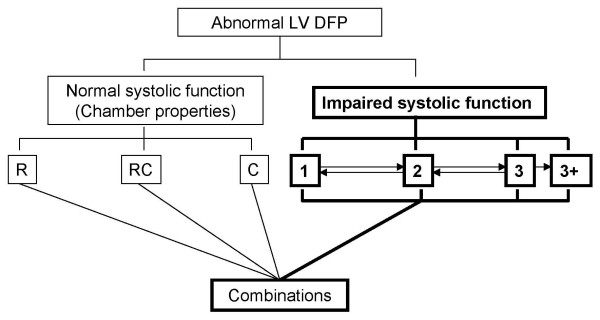
**Classification of Doppler filling patterns in cardiac pathologies**. Algorithm to follow for cardiac causes of abnormal left ventricular Doppler filling pattern. The deciding point here is the systolic function. When the systolic function is normal the distinctive patterns are R, C and RC. In this case one pattern does not 'progress' to another. In systolic dysfunction there is a seamless progression of patterns from 1 – 3+ as the systolic function worsens. These can progress in the reverse direction from 3 – 1 when systolic function improves.

The above classification is based on currently available information. A deductive reasoning and logical extrapolation has been used to arrive at the conclusions. The scientific method of 'elimination of variables' should be applied as follows to derive meaningful information (see table 1). When confronted with an abnormal LV DFP the following steps may be taken for proper analysis: Once it is technically proper and physiological factors are eliminated, the key question to ask is what is the systolic function? (see figure 6)?

**Table 1:** Common variables that affect Doppler patterns.

**Technical factors**
Image quality
Position of sample volume
**Physiological factors**
Heart rate
Respiration
Position
Preload
Valsalva maneuver
Age

**Pathological causes**
Non-cardiac
Obesity
Deconditioning
Chronic obstructive airways disease
Sleep apnoea
Renal failure
Cardiac
See Table 2 and Figure 6

Each of the factors mentioned could confound Doppler interpretation. So these factors should be eliminated or their effects should be factored in. The list is not exhaustive and the non-cardiac causes could also affect cardiac function.

If the LV systolic function is good then it has some diagnostic role: In that case is it an R pattern? (hypertension, ischaemia, diabetes or hypertrophic cardiomyopathy), a C pattern? (restrictive cardiomyopathy or pericardial disease), or a combination RC pattern? The R pattern could be age related and non-cardiac pathologies like obesity, sleep apnoea, deconditioning or renal failure could produce patterns between 0 and 1.

If the systolic function is not satisfactory, then one can assess the severity and prognosticate using the LV DFP as described above. In cases with wall motion abnormality with apparently normal LV contractility a pattern to the right of 0 could signify early systolic dysfunction. Remember, *LV DFP can only be interpreted in the context of systolic function.*

In a complex situation, it is possible to identify the problem by recognizing the predominant hemodynamic pattern and factoring in the complicating chamber property.

The accuracy of interpretation of LV DFP is best in the young (<50 years) where age related changes due to atrial, ventricular and valvular stiffness are eliminated.

**Figure 7:**
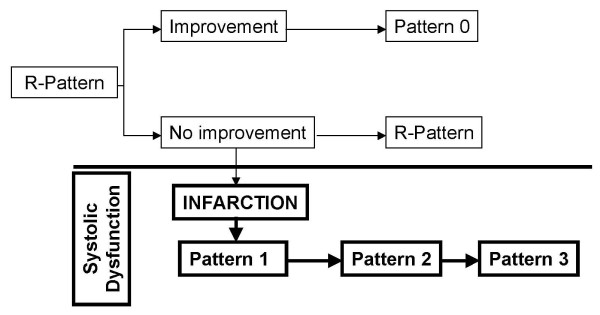
**Progression of LV DFP in disease**. Diagram shows pattern progression in hypertension. Hypertension of sufficient duration with preserved systolic function causes an R pattern. If there is improvement the pattern could revert to normal. If there is no improvement the pattern could remain as R pattern with probably an increase in EFT. With preserved systolic function it will never progress to C pattern – implying another disease. However in the event of a myocardial infarction *systolic dysfunction *occurs and the patterns will progress from 1 to 3+ depending on the severity of systolic dysfunction. Here it is better not to call it a progression of 'diastolic dysfunction'.

A clinical example will demonstrate the use of this methodology. Consider a 35 year old patient with myocardial infarction. There was apparently acceptable eye-ball LV function with one akinetic apical segment. The trans-mitral Doppler showed E<A with a normal EFT. This is pattern 1 and could signify a predominantly mild LV systolic dysfunction rather than 'diastolic dysfunction'. A practical example of pattern progression in disease state is shown in figure 7.

## Implications of the hypothesis

Transmitral Doppler patterns are not just useful for evaluating diastolic function. They are also useful to evaluate systolic function. By following this schema, it will be possible to fit these patterns into specific groups thus aiding in diagnosis and treatment. The detection of R pattern should make the clinician search aggressively for causes like hypertension, diabetes, ischaemic heart disease and cardiomyopathy in the young (below 50 years of age). Similarly, the detection of pattern 1 in a young individual could also indicate *systolic *dysfunction and should make the clinician search diligently for wall motion abnormalities. DFP could also indicate prognosis.[21,22]

Doppler patterns are easy to acquire and are intriguing. However the interpretation is difficult. This methodology presents a gross simplification of a complex phenomenon. But by following this systematic analysis we could get useful diagnostic information and therapeutic options. This methodology uses pattern recognition with minimal measurements. The only measurement of EFT can also be done away with once the operator is used to the display sweep settings. It is practical and can be applied in day to day clinical practice. The precise terms will improve communication and standardize research.

## Abbreviations

LV: Left ventricle; DFP: Doppler filling pattern; R-pattern: Relaxation abnormality pattern; C-pattern: Compliance abnormality pattern; RC-pattern: Combination of R and C patterns; EFT: Early filling time; LAP: Left atrial pressure.

## Competing interests

The author declares that they have no competing interests.

**Table 2:** Examples of cardiac causes of abnormal LVDFP

**Normal systolic function**
R Pattern
Hypertension
Ischaemia
Diabetes
Hypertrophic cardiomyopathy
C Pattern
Pericardial disease
Restrictive cardiomyopathy
RC Pattern
Hypertension with amyloidosis
**Abnormal systolic function**
Patterns to the right of '0'
Ischaemia
Dilated cardiomyopathy
Myocarditis etc

Table shows the commonly encountered examples of cardiac causes of abnormal LVDFP. The list is not exhaustive. Any cardiac condition could cause abnormal LVDFP when it is significant enough to affect Doppler. For example, right sided pathologies would affect LVDFP.
